# Supplementary material for: RNA-binding proteins regulate aldosterone homeostasis in human steroidogenic cells
Source: RNA. 2021 Aug;27(8):933–45. doi: 10.1261/rna.078727.121 (PMC8284322; doi:10.1261/rna.078727.121)
Supplement: Supplemental Material [file supp_27_8_933__DC1.html]

RNA-binding proteins regulate aldosterone homeostasis in human steroidogenic cells — Supplemental Material 

# RNA-binding proteins regulate aldosterone homeostasis in human steroidogenic cells

## Supplemental Material

- Supplemental\_Figures\_.pdf
- supplemental\_methods.pdf
- Supplemental\_Table\_1.zip
- Supplemental\_Table\_2.zip
- Supplemental\_Table\_3.zip
- Supplemental\_Table\_4.zip
- Supplemental\_Table\_5.zip
- Supplemental\_Table\_6.zip
- Supplemental\_Table\_7.zip
- Supplemental\_Table\_8.zip
- Supplemental\_Table\_9.zip
- Supplemental\_Tables\_.docx
