# Supplementary material for: RNA-binding proteins regulate aldosterone homeostasis in human steroidogenic cells
Source: RNA. 2021 Aug;27(8):933–45. doi: 10.1261/rna.078727.121 (PMC8284322; doi:10.1261/rna.078727.121)
Supplement: Supplemental Material [file supp_078727.121_Supplemental_Figures_.pdf]

SUPPLEMENTAL FIGURES

RNA-binding proteins regulate aldosterone homeostasis in human steroidogenic cells

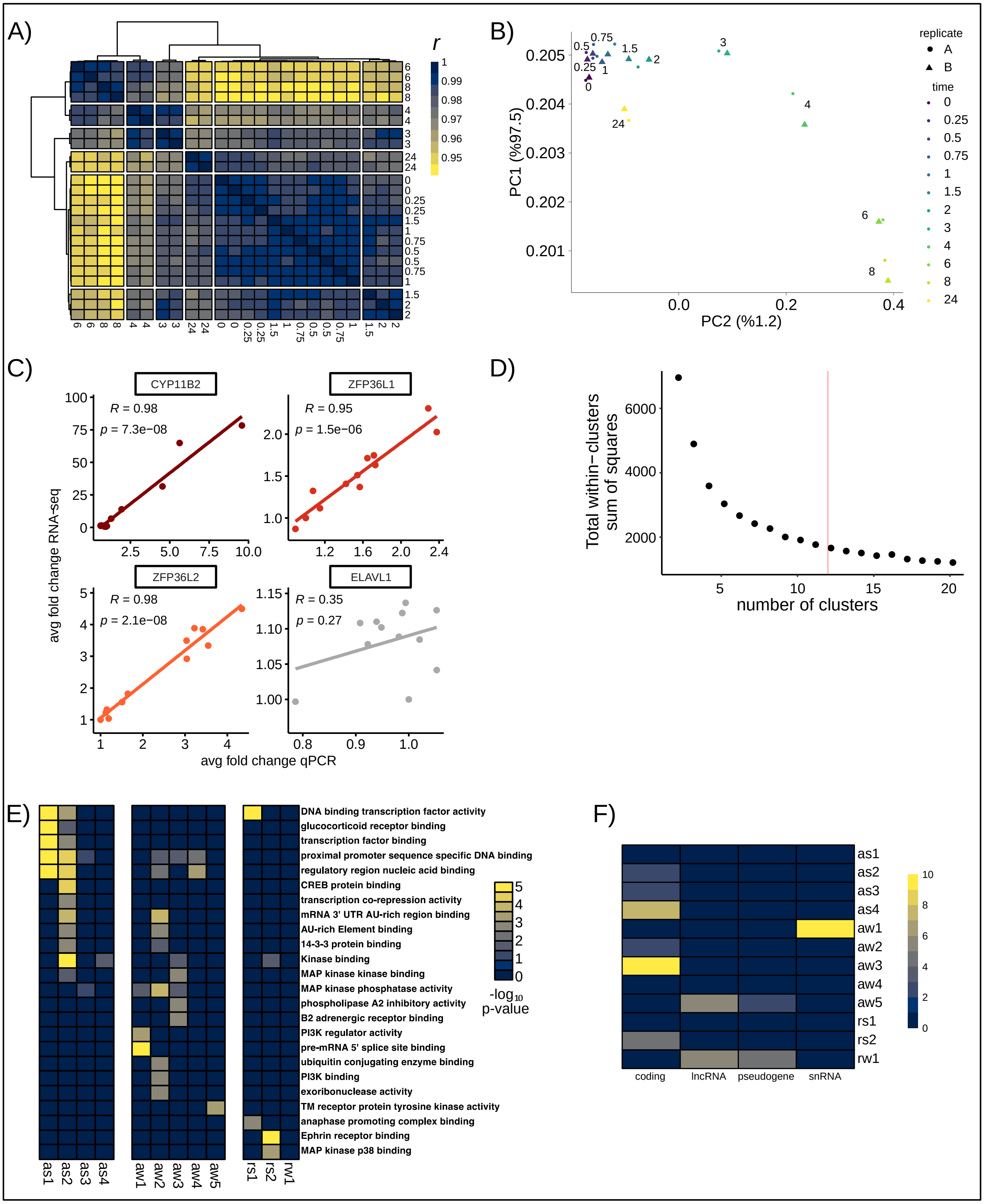

Supplemental Figure 1 – AngII stimulation RNA-seq time course. A) Heatmap of clustered pairwise Pearson correlation coefficients for all samples using expressed genes. B) PCA analysis of all samples using expressed genes. C) Comparison of fold change in expression relative to unstimulated H295R cells between RNA-seq (y-axis) and qRT-PCR. D)Determination of the number of clusters. E) Heatmap of gene biotypes that are significantly enriched in the AngII response groups. F) Heatmap of selected gene sets (Biological Process ontology) that are significantly enriched in the AngII response groups.

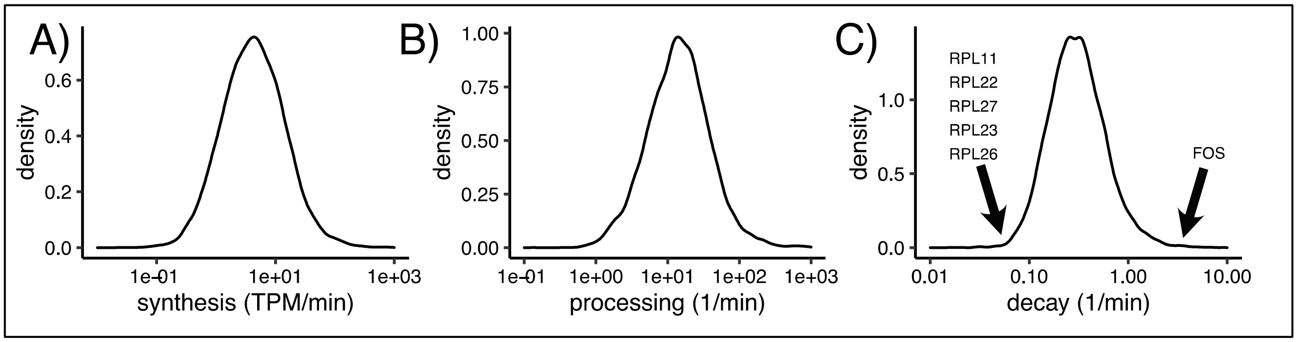

Supplemental Figure 2 – Measuring RNA decay rates in unstimulated H295R cells. Distribution of A) synthesis, B) processing, and C) decay rates calculated using INSPeCT comparing 20-minute 4sU pulse to input RNA. Decay rates of mRNAs encoding ribosomal proteins and immediate early genes depicted.

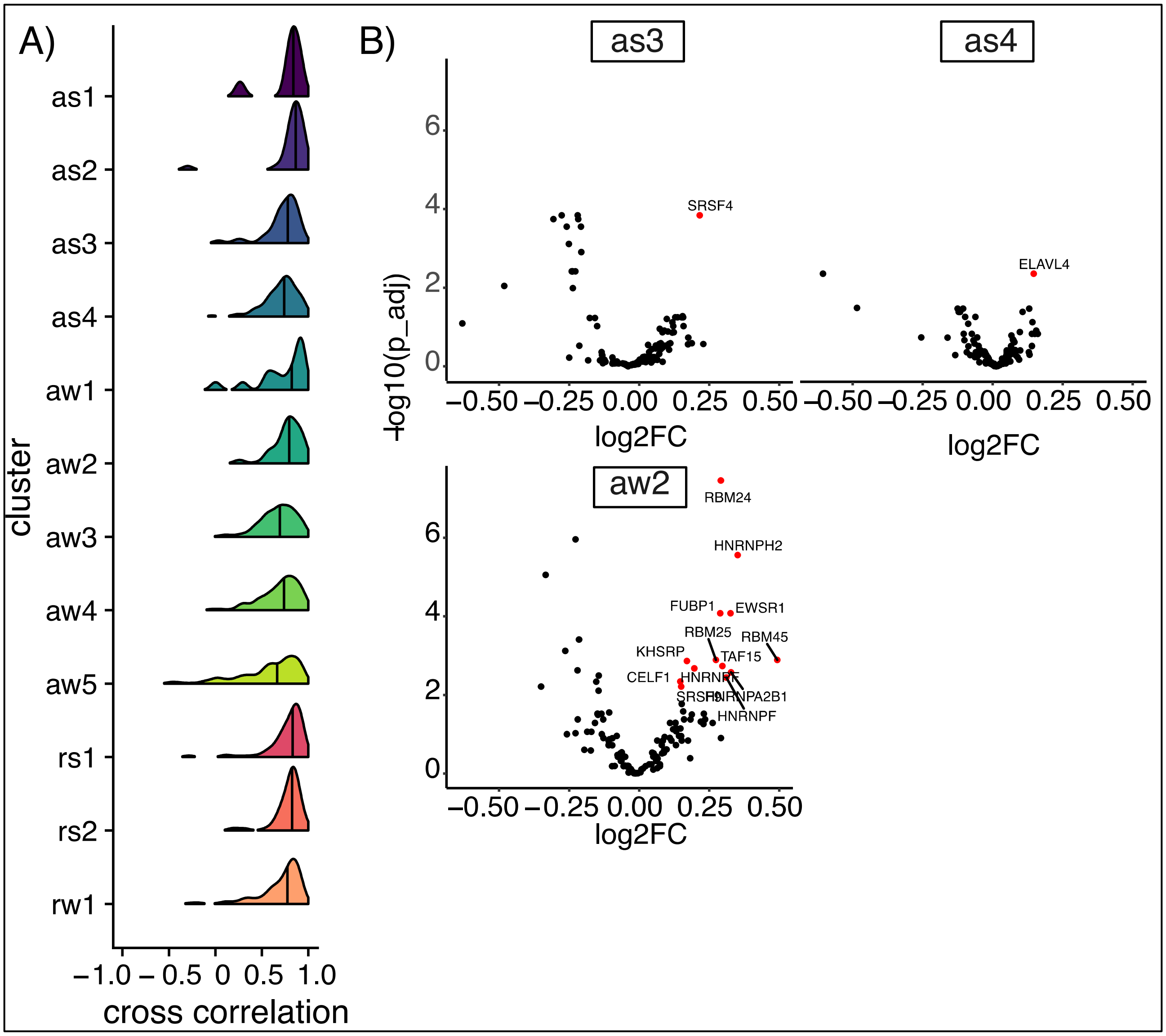

Supplemental Figure 3 – AngII-induced changes in RNA decay. A) Distribution of cross-correlation coefficients between pre-mRNA and mRNA levels for each gene across all time points for each response cluster. B) Scatter plot of the p-value and enrichment of RBP motifs in 5mers within the 3' UTR of destabilized mRNAs in as3 (top left), as4 (top right), and aw2 (bottom left) versus 3' UTRs of non-differentially expressed mRNAs.

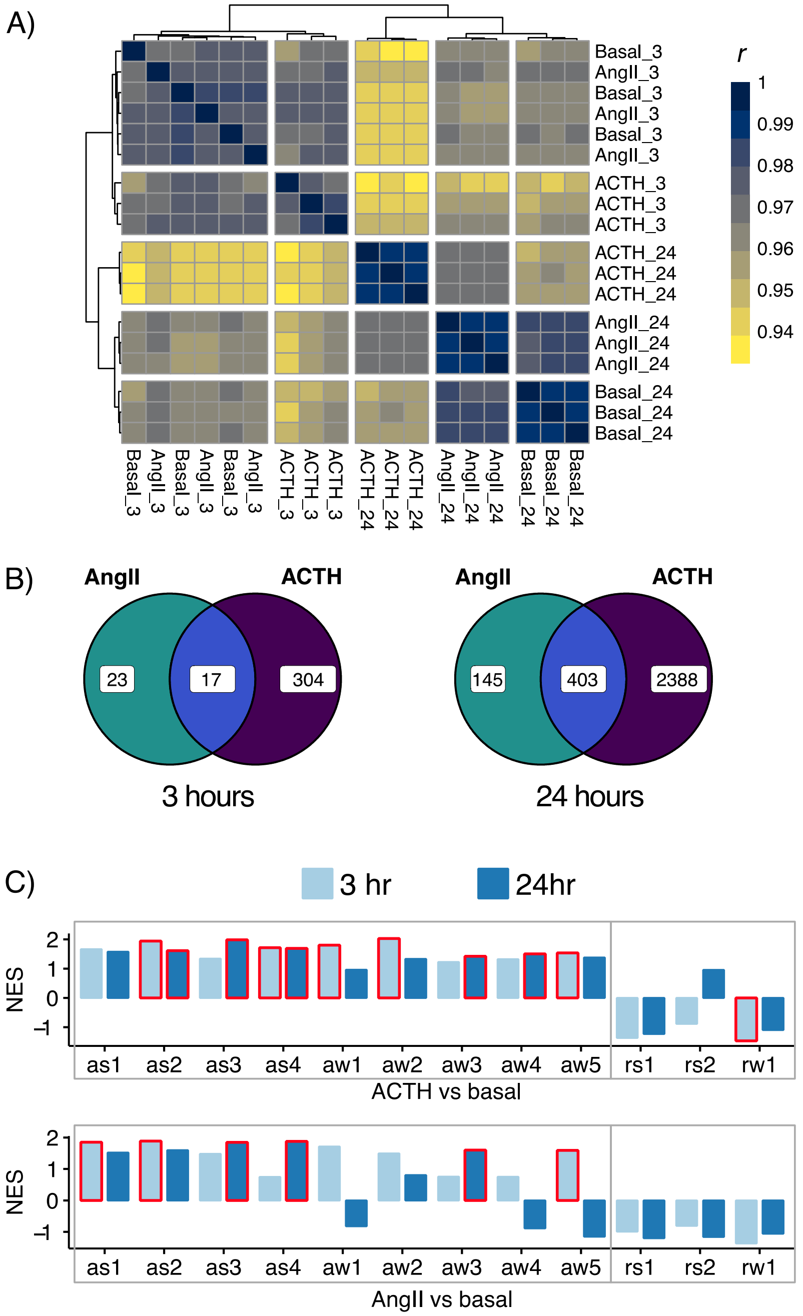

Supplemental Figure 4 –Ex vivo stimulated primary human adrenocortical cells analysis. A) Heatmap of clustered pairwise Pearson correlation coefficients for all samples using expressed genes. B) Venn diagram depicting union and differences between genes differentially expressed upon ex vivo stimulation of primary human adrenocortical cells with AngII or ACTH for 3 hours (left) or 24 hours (right). C) Barplot of normalized enrichment scores (NES) calculated using GSEA representing the enrichment (positive values) or depletion (negative values) of gene sets identified from H295R stimulation response in ACTH (top) or AngII (bottom) induced changes in expression versus basal for each time point. The NES is comparable across gene sets since it adjusts for the size of the gene set and represents the strength of the enrichment/depletion. H295R-derived gene sets with statistically significant enrichment or depletion ($p < 0.05$) outlined in red.

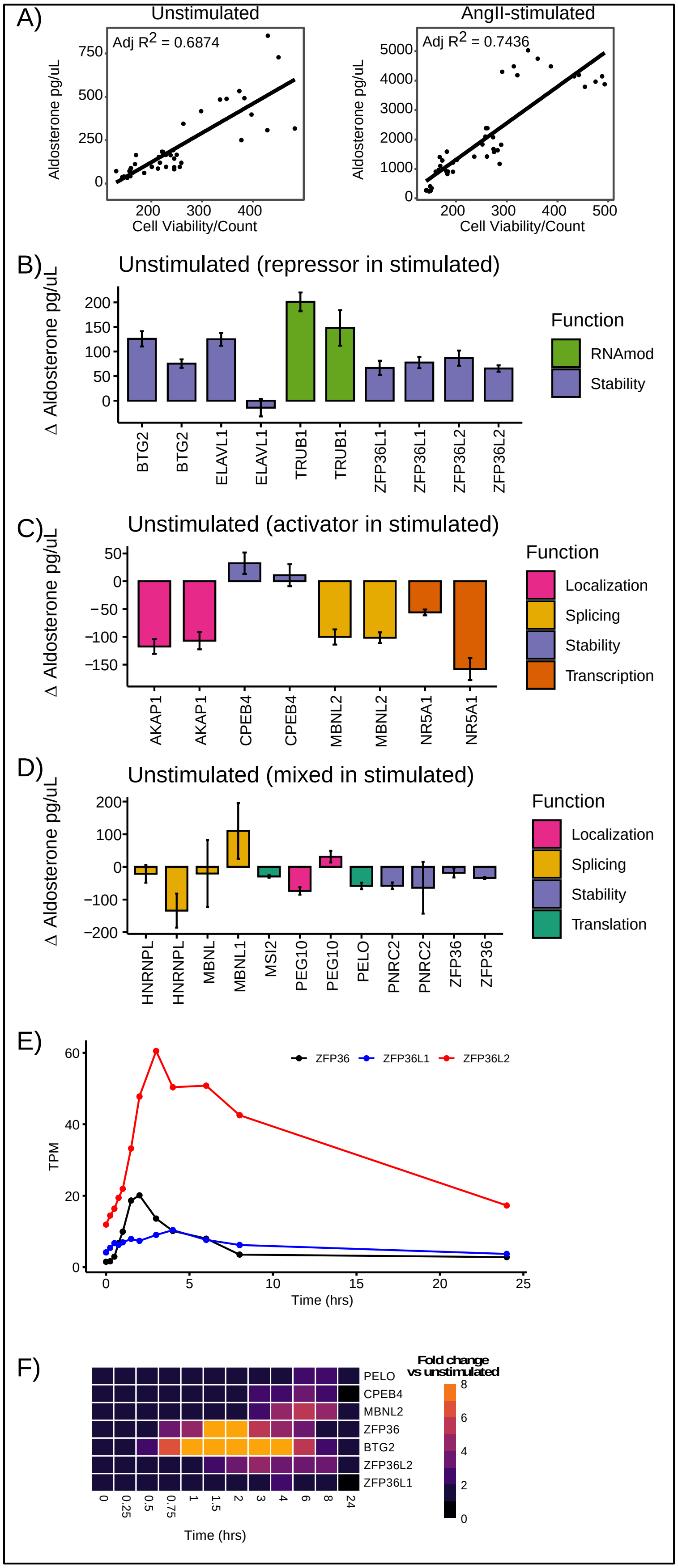

Supplemental Figure 5 – **Regulation of aldosterone by RBPs in unstimulated cells.** A) Scatterplot of aldosterone levels (supernatant) to cell viability measured using PrestoBlue for unstimulated (left) and AngII-stimulated cells (right). To aid comparison with aldosterone changes from the AngII stimulated cells (Figure 5B-D), the plot arrangement and category assignments ("repressor", "activator", "mixed") are identical to the simulated results. Barplot of the change in aldosterone concentrations for knockdowns resulting in B) increased aldosterone concentration, C) decreased aldosterone concentration, and D) discordant or single siRNA results. Aldosterone levels were measured using ELISA from supernatants of H295R cells electroporated with siRNAs targeting candidate RBPs. The y-axis represents the change in aldosterone concentration versus mock electroporation (see methods). The error bars represent the standard error of at least 6 replicates. RBPs are color-coded by their known function. E) Expression levels in transcripts per million (TPM) of all three ZFP36 family members during the AngII stimulation time course. F) Heatmap depicting change in expression compared to unstimulated cells for mRNAs encoding proteins screened for regulation of aldosterone. Only those with statistically significantly differential expression were included.

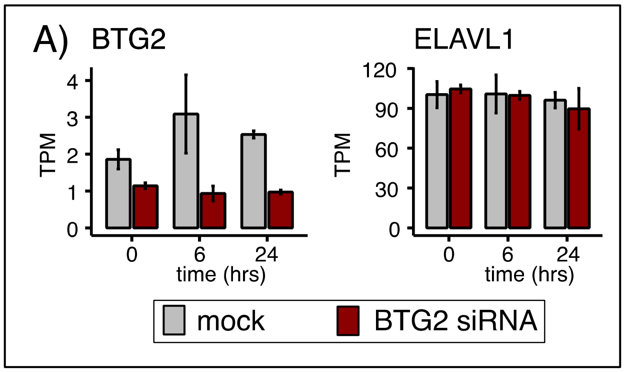

Supplemental Figure 6 – **BTG2-dependent steroidogenic expression changes.** A) Barplot of expression levels of BTG2 (downregulated, left) and ELAVL (no change, right) upon BTG2 siRNA depletion and AngII stimulation.
