# Supplementary material for: RNA-binding proteins regulate aldosterone homeostasis in human steroidogenic cells
Source: RNA. 2021 Aug;27(8):933–45. doi: 10.1261/rna.078727.121 (PMC8284322; doi:10.1261/rna.078727.121)
Supplement: Supplemental Material [file supp_078727.121_Supplemental_Tables_.docx]

**SUPPLEMENTAL TABLES**

Supplemental Table 1 - AngII-treated H295R mature RNA abundance quantification

Supplemental Table 2 - AngII-treated H295R mature RNA k-means clustering, differential gene expression analysis, and correlation results

Supplemental Table 3 - Estimated decay rates in for unstimulated H295R

Supplemental Table 4 - AngII-treated H295R precursor RNA abundance quantification

Supplemental Table 5 - Exon-intron-split analysis results

Supplemental Table 6 - AngII-treated adrenocortical cell mature RNA abundance quantification and differential gene expression analysis

Supplemental Table 7 - ACTH-treated adrenocortical cell mature RNA abundance quantification and differential gene expression analysis

Supplemental Table 8 - Changes in aldosterone levels from siRNA screen.

Supplemental Table 9 - BTG2 knockdown mature RNA abundance quantification
